# Functional brain activity in persistent postural-perceptual dizziness (PPPD) during galvanic vestibular stimulation reveals sensitization in the multisensory vestibular cortical network

**DOI:** 10.1038/s41598-025-11529-2

**Published:** 2025-07-27

**Authors:** Renana Storm, Viktoria Wrobel, Antonia Frings, Andreas Sprenger, Christoph Helmchen

**Affiliations:** 1 Department of Neurology, University Hospital Schleswig-Holstein, Lübeck, Germany; 2 Center of Brain, Behavior and Metabolism (CBBM), University of Lübeck, Ratzeburger Allee 160, 23562 Lübeck, Germany; 3 Institute of Psychology II, University Lübeck, Lübeck, Germany

**Keywords:** Vestibular perception, PPPD, GVS, fMRI, Persistent postural-perceptual dizziness, Magnetic resonance imaging, Neuroscience

## Abstract

Persistent postural-perceptual dizziness (PPPD) is often preceded by vestibular disorders. We applied galvanic vestibular stimulation (GVS) and related stimulus-evoked activity to individual ratings of perceived motion for each stimulus and to perceived egomotion thresholds by GVS and behavioural parameters outside the scanner: levels of functional disability by standardized questionnaires, visual motion coherence, passive egomotion perception by chair rotation and quantitative postural stability. We hypothesized that the preceding vestibular disorder predisposes to abnormal brain excitability by vestibular stimulation. All participants showed normal vestibular function tests on quantitative testing. GVS with different intensities was applied to 28 patients and 28 age- and gender-matched healthy participants (HC) in the scanner. After each stimulus, participants rated their perceived level of egomotion. GVS perception threshold was significantly lower in PPPD patients. Contrasting stimulus-identical GVS against a sham stimulus, group comparison revealed a stronger activation in the patient’s supramarginal gyrus, insular cortex (operculum 3), and vermis. This stronger excitability was not related to the individual threshold of perceived egomotion by GVS. Patients rated GVS-evoked egomotion intensity by identical GVS intensities larger than HC but neural activity did not correlate with individual ratings of perceived egomotion by GVS. As GVS evoked larger egomotion and larger brain activation in patients, the ratio of brain activity to egomotion perception was not different between groups. GVS-evoked insular activity increased with the level of PPPD-related disability and postural imbalance. The larger activation in multisensory cortical vestibular network indicates a sensitization to vestibular stimuli eliciting egomotion perception which increases with levels of PPPD disability. It seems to reflect a sensory-neural amplification rather than an abnormal sensory-perceptual scaling.

**Supplementary Information:**

The online version contains supplementary material available at 10.1038/s41598-025-11529-2.

## Introduction

According to the classification criteria of the Bárány Society in 2017, persistent postural-perceptual dizziness (PPPD) is a chronic disorder with dizziness and perceived unsteadiness for at least 3 months, with exacerbation by upright posture, active or passive self-motion, and exposure to environments with complex or moving visual stimuli, that is not proficiently explained by another disorder^1^. PPPD often occurs secondary due to preceding destabilizing disorders, such as central or peripheral vestibular disorders, migraine, prolonged physiological multisensory stimulation (Mal de débarquement syndrome)^2^ anxiety disorders or adverse reactions to medications^1,3,4^.

As PPPD severity is not related to the magnitude of previous or still persistent vestibular dysfunction^5^ brain sensitization in response to the preceding stimuli is a feasible mechanism but has not been examined yet. Potential mechanisms of this maladaptation include: (i) predictive processing of sensory inputs by abnormal bottom-up central processing of self and external motion signals, (ii) misprediction of the sensory consequence of one‘s own movements (efference copy) and (iii) alterations in motion perception that influences top-down postural control^6–8^. A mismatch between ‘bottom-up’ (vestibular/proprioceptive sensory) inputs and maladaptive signals from ‘top-down’ attentional control systems has been suggested to determine perceived postural imbalance^7^. One trigger may come from altered thresholds of sensory (visual, vestibular, proprioceptive) motion perception.

PPPD patients preferably seem to rely on visual inputs for balance control compared to other sensory, i.e., vestibular and somatosensory inputs^7,9^. Sensitivity to moving visual stimuli often becomes annoying in PPPD. This may be related to an abnormal sensitivity in the visual cortex, e.g., in response to virtual reality moving scenes^10,11^. This visual dependence resembles functional neural reorganization in patients who suffered from unilateral vestibulopathy with subsequently developing abnormal visual impact on vestibular perception during the course of the disease^12,13^. We recently demonstrated poorer visual motion detection in PPPD, i.e., elevated visual motion coherence thresholds^14^.

Vestibular motion perception thresholds are lower during binaural galvanic vestibular stimulation in PPPD^14,15^ but not during rotatory vestibular stimulation around the earth-vertical axis^14,16^. It is unknown, whether and how altered sensory (e.g., visual, vestibular) thresholds of motion perception lead to functional changes in the responsivity of core vestibular brain regions to experimental sensory stimuli.

Tasked-based (i.e., sound-evoked vestibular stimulation, visual motion stimuli) and resting functional magnetic resonance imaging (fMRI) studies have been used to investigate altered brain activation and functional connectivity (FC) in PPPD patients (or its precursor conditions). Sound-evoked vestibular stimulation of the otoliths in the PPPD precursor condition “chronic subjective dizziness” revealed a lower activation of the parieto-insular vestibular cortex (PIVC), the anterior and posterior insula, and the anterior cingulate cortex (ACC)^17^. These brain regions belong to the multisensory vestibular network^18–20^ receiving vestibular, visual and somatosensory inputs which are crucial for spatial orientation, balance and postural control.

The greater reliance on visual compared to vestibular signals for achieving spatial orientation and maintaining balance in PPPD was suspected to derive from altered cortical visual processing^6,10,21–23^. Visual cortex activity increased during simulated vertical visual motion in PPPD in proportion to the dizziness handicap^10^ but was found to be normal in a related study with various forms of complex visual stimulation by checkerboard and optokinetic stimulus patterns^24^. During visual motion simulation, patients with phobic postural vertigo showed a larger activation in the sub-genual anterior cingulate cortex (ACC)^25^. Resting state fMRI studies in PPPD precursor conditions showed a reduced functional connectivity in the hippocampus with the operculum, the insular cortex and the cerebellum^21,26^. The role of personality traits in PPPD became overt as neuroticism increased with the neural activity and connectivity of neural networks that mediate attention to vertical visual motion^23^.

Unlike sound-evoked vestibular stimulation^17^ galvanic vestibular stimulation (GVS) provides semicircular canal stimulation and elicits egomotion perception, altered perception of verticality^27–29^ and postural imbalance^15,30^. However, it has not been applied in PPPD patients yet to study vestibular brain responsivity despite the fact that abnormal egomotion perception is a core feature in PPPD. As GVS neither induces nausea or vomiting nor head movements it is suitable for fMRI studies.

Therefore, we compared GVS-evoked brain activity (contrasted against sham stimulation) in PPPD patients and healthy participants (HC) and related it to the individual egomotion thresholds by GVS, the ratings of perceived egomotion intensity by a suprathreshold GVS, and disease related parameters (duration, levels of functional disability by standardized questionnaires), as well as individual parameters obtained outside the scanner: visual motion coherence, passive egomotion perception by chair rotation and quantitative postural stability.

We hypothesized that (i) PPPD patients perceive and rate GVS intensity higher than HC, (ii) GVS induces larger brain activity in the multisensory vestibular network, which (iii) relates to perceptional and behavioural parameters supporting disease-linked mechanisms.

## Results

### Participants

The mean age of the patients was 43.3 ± 11.4 years and 43.4 ± 12.6 years for HC. The mean disease duration was 34 ± 26 months. Elevated NPQ and ALQ scores were only present in patients as expected (NPQ: t(53) = 10.42; ALQ: t(54) = 11.71, *p* < 0.001 for both). Patients scored significantly larger values for the HADS (HADS-Anxiety: t(54) = 4.76; HADS-Depression: t(54) = 4.40, *p* < 0.001 for both) and for the neuroticism trait of the NEO-FFI (Mann-Whitney-U: Z = −3.73, *p* < 0.001), whereas the extraversion trait (*p* = 0.075) and the MSSQ (*p* = 0.136) did not differ between groups. The current quality of life was significant lower in patients (Mann-Whitney-U: Z = −5.24, *p* < 0.001). For details see^14^. Patient’s individual GVS threshold was significantly lower than in HC (patients: 0.28 ± 0.11 mA, HC: 0.42 ± 0.16 mA; t(54) = −4.10, *p* < 0.001). Patients rated *low* and *fix* GVS intensity higher than HC (see Table 1).

**Table 1:** Perceived intensities of egomotion elicited by GVS.

GVS intensity	PPPD patients scores (%)	HC scores (%)	p-value
Sham	14.63 ± 12.81	07.66 ± 10.16	0.116
Low	29.32 ± 17.25	18.47 ± 9.94	**0.007**
Fix	48.31 ± 19.80	31.70 ± 16.38	**0.001**
High	63.01 ± 20.74	54.66 ± 19.38	0.183

PPPD and HC ratings of the 4 GVS intensities: *low* GVS, *fixed* GVS and *high* GVS and *sham* GVS (range 0-100%) with group comparison with corresponding p-values (significant p-values are marked bold). GVS = galvanic vestibular stimulation, PPPD = persistent postural-perceptual dizziness, HC = healthy control subject.

### Imaging results

#### Stimulus related brain activity

**Fig. 1:**
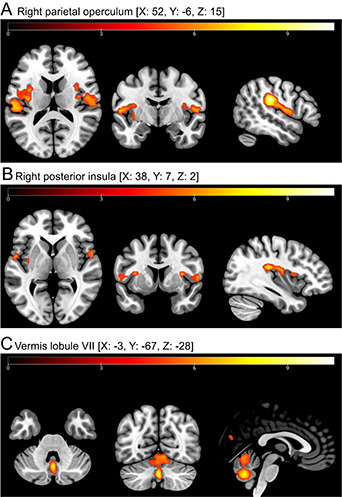
GVS-evoked brain activation (main effect, contrasting *fixed* GVS against the *sham* condition) in the bilateral parietal operculum (**A**), posterior insula (**B**) and lobule VII of the vermis (**C**) in PPPD patients and HC. Axial, coronal and sagittal view (from left to right), irrespective of the group (patient/HC). The coordinates (x, y, z) are in the Montreal Neurological Institute space. Whole brain analysis was performed using FWE-correction (*p* < 0.05). Tvalues are indicated along the colour bars. Abbreviations: HC = healthy control subject, PPPD = persistent postural-perceptual dizziness.

Using whole brain analysis, there was a main effect for STIMULUS (*fixed* GVS, *low* GVS, *high* GVS, contrasted against the *sham* condition) in both patients and healthy control subjects, i.e., brain activation increased with stimulus intensity in several multisensory cortical areas including those known to be associated with vestibular processing, i.e., in the parietal operculum (OP) bilaterally, posterior insula, vermis (ventral lobule VII) and the uvula (FWE *p* < 0.05) (Fig. 1A-C).

#### Group differences

There was a main effect for GROUP. There was a larger activation in the vermis (ventral lobule VII), the right lobule IV and V of the cerebellar hemisphere, the left superior parietal lobe, the left paracentral lobule and the right supplementary motor area of patients across all GVS intensities (see Supplementary Table S1) while the opposite contrast (HC > patients) did not reveal significant activations (FWE corrected, *p* < 0.05). The interaction between GROUP x STIMULUS revealed larger brain activation primarily in vestibular and visual brain regions of patients (see Supplementary Table S1) for *low* GVS (*p* < 0.001 uncorrected), *fix* and *high* GVS (FWE corrected, *p* < 0.05).

#### Region of interest analyses

**Fig. 2:**
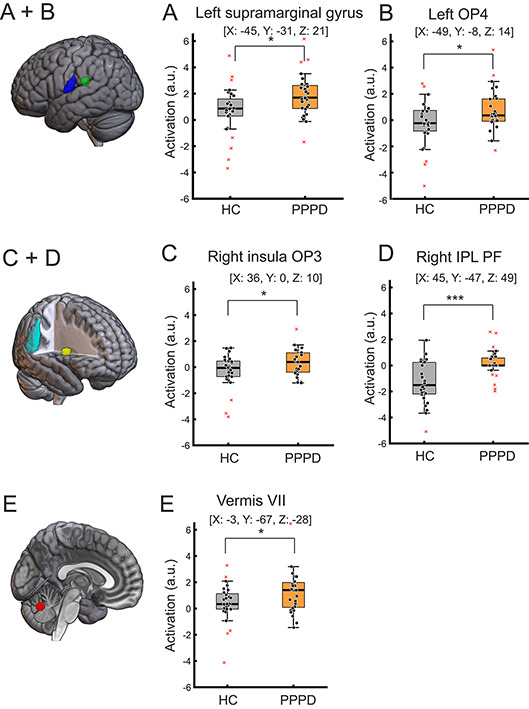
Group comparisons (PPPD vs. HC) using ROI analysis for brain activations during *fixed* GVS. *Fixed* GVS elicited stronger activation in the patients’ supramarginal gyrus (**A**), the left operculum (OP4) (**B**), the right insula OP3 (**C**), the right IPL PF (**D**) and lobule VII of the vermis (**E**). The coordinates (x, y, z) are in the Montreal Neurological Institute space. Error bars indicate standard error of mean [ROI analysis, FWE *p* < 0.05 corrected]. * ≤ 0.05, *** < 0.001. Abbreviations: HC = healthy control subject, IPL PF = right inferior parietal lobe, OP3/4 = parietal operculum, PPPD = persistent postural-perceptual dizziness.

Following the enhanced brain activity in PPPD patients, we performed region of interest (ROI) analyses in brain areas known to be involved in human egomotion^19^ and the visual cortex due to its reciprocal interactions^31^. *Fixed* GVS elicited a stronger activation in the supramarginal gyrus (t(54) = −2.50; *p* = 0.016, Fig. 2A), the left OP4 (T(54) = −2.14, *p* = 0.037, Fig. 2B), the insular operculum region OP3 (t(54) = −2.16, *p* = 0.035, Fig. 2C), the right inferior parietal lobe (IPL PF) (t(54) = −2.63; *p* = 0.011, Fig. 2D) and in the lobule VII of the patients’ vermis (t(54) = −2.32; *p* = 0.024, Fig. 2E). There was no group difference particularly regarding the visual area of the cingulate sulcus (CSv) (*p* = 0.343), the uvula (*p* = 0.056), the posterior and anterior insula (p always > 0.132) and the visual areas V1 – V5 (p always > 0.069). *High* GVS and *low* GVS revealed no significant activation difference (p always > 0.146). There was no significant activation in the reverse contrast (HC > patients). A list of all used ROI labels and their corresponding name are listed in Supplementary Table S2.

**Fig. 3:**
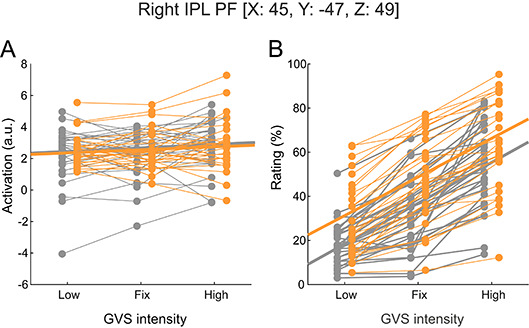
Right IPL brain activation (**A**) and rating (**B**) for GVS intensities *low*, *fix* and *high* between PPPD patients and HC. Vestibular brain activation does not increase with enhanced GVS intensity, exemplarily shown in the right inferior parietal lobe. Patients perceived GVS-evoked egomotion (rating in %) significantly higher than HC for *low* and *fix* GVS with an overall increase of egomotion perception with increasing GVS intensity for both patients and HC. Patients are marked orange, HC grey. The coordinates (x, y, z) are given for the Montreal Neurological Institute space. Abbreviations: GVS = galvanic vestibular stimulation, HC = healthy control subject, IPL PF = right inferior parietal lobe.

Within these ROIs, increased GVS-intensity did not elicit higher brain activation (p always > 0.103, Fig. 3A). ROI activation was not correlated with the GVS threshold, neither in patients nor in HC. Using an Analysis of Covariance (ANCOVA) with the individual GVS threshold as a covariate there was still increased brain activity in PPPD patients indicating that this increase is independent of the lower perception threshold of patients (see Supplementary Table S3). GVS-evoked ROI activation did not correlate with the ratings of perceived egomotion intensity by GVS, neither for *low* GVS (patients: p always > 0.064, HC: p always > 0.587), *fixed* GVS (patients: p always > 0.198, HC: p always > 0.177) or *high* GVS (patients: *p* > 0.319, HC: p always > 0.293, contrary to the ratings of perceived egomotion itself (Fig. 3B). The calculated ratio between GVS-evoked brain activity and perceived egomotion intensity did not differ between PPPD and HC (p always > 0.3). GVS-evoked brain activity was not correlated with disease duration for any GVS intensity, except for the right OP2 during *high* GVS (*p* = 0.028, *r* = −0.42). There was no correlation of brain GVS-evoked activation of HC for any ROI with any GVS intensity.

**Fig. 4:**
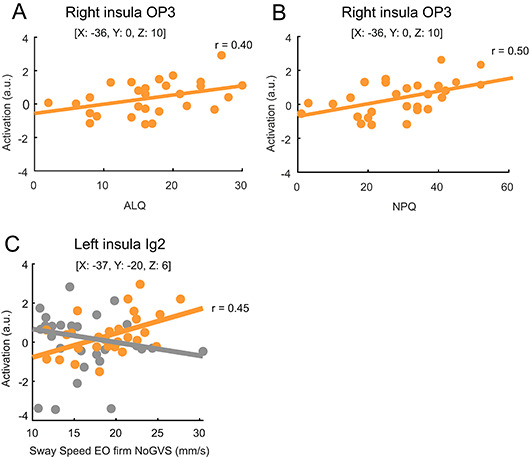
Insular cortex activation of OP3 by *fixed* GVS increases with level of PPPD-related functional disability (**A**: ALQ, **B**: NPQ) and postural sway speed in the simple platform condition (eyes open [EO], firm surface, no GVS, **C**). Patients are marked orange, HC grey. The coordinates (x, y, z) are in the Montreal Neurological Institute space. Abbreviations: ALQ = Athens-Lubeck-Questionnaire, EO = eyes open, GVS = galvanic vestibular stimulation, HC = healthy control subject, Ig2 = part 2 of the granular insula, OP3 = parietal operculum, NPQ = Niigata PPPD Questionnaire.

*Fixed* GVS-evoked activation increased with PPPD-related dizziness severity in the insular operculum (OP3) (NPQ: *p* = 0.006, *r* = 0.50; ALQ: *p* = 0.036, *r* = 0.40, Fig. 4A + B), in the bilateral OP4 (NPQ: *p* = 0.028, *r* = 0.42 for left and *p* = 0.012, *r* = 0.47 for right; ALQ: *p* = 0.026, *r* = 0.42 for left and *p* = 0.032, *r* = 0.41 for right) and in the right V1 and V2 (NPQ: *p* = 0.018, *r* = 0.44 for V1 and *p* = 0.026, *r* = 0.042 for V2, ALQ: *p* = 0.018, *r* = 0.44 for V1 and *p* = 0.021, *r* = 0.43 for V2). In addition, patients’ activation (*fixed* GVS) increased with ALQ alone in the left insula (*p* = 0.025, *r* = 0.42), in the cerebellar lobule VII (*p* = 0.018, *r* = 0.44) and in the uvula of the vermis (*p* = 0.023, *r* = 0.43). Even activation by *low* GVS increased with ALQ in the insula OP3 (NPQ: *p* = 0.011, *r* = 0.47; ALQ: *p* = 0.015, *r* = 0.45), bilateral OP4 (ALQ: *p* = 0.036, *r* = 0.40 for the left and *p* = 0.034, *r* = 0.40 for the right), lobule VII of the vermis (NPQ: *p* = 0.022, *r* = 0.43; ALQ: *p* = 0.003, *r* = 0.55) and in the right CSv (*p* = 0.010, *r* = 0.48). *Fixed* GVS and *high* GVS-evoked activation in the primary visual cortex (V1, V2 bilaterally and the left V5) decreased with values of the extraversion trait of the NEO-FFI (see Supplementary Table S4).

There were no significant correlations for brain activation by any GVS with any other questionnaire parameters (MSSQ, EQ-5D-3 L, HADS, other traits of the NEO-FFI).

Finally, GVS-evoked activation in any ROI was not correlated with the individual visual motion perception threshold (p always > 0.45) or passive egomotion threshold (p always > 0.113). GVS-evoked activation (*fixed* GVS) increased with the postural imbalance (postural sway speed with the eyes open while standing on a firm platform^30^, in the two parts of the granular insula Ig1 (*p* = 0.032, *r* = 0.41) and Ig2 (left hemisphere; *p* = 0.016, *r* = 0.45; Fig. 4C). This was not the case in the eyes closed condition (p always > 0.139). GVS-evoked brain activation in HC did not show any significant correlation with GVS intensities or any other behavioral or perceptual parameter.

## Discussion

Vestibular stimulation (GVS) reliably elicited activation in core regions of the vestibular network known to be involved in vestibular processing and egomotion perception: the parietal and insular operculum, posterior insula and the cerebellum, specifically the uvula^18,19,32,33^. This network is multisensory and receives vestibular, visual and somatosensory inputs. Vestibular signals conveying head and body motion are aligned with visual motion signals, provided by projections from MT/V5 to OP2^34^ the core vestibular processing region^18^ to ensure egomotion perception. GVS activates the peripheral vestibular system, forwarding its information mainly to temporo-parietal regions (parietal operculum [right OP2]) and posterior insula for motion perception and spatial orientation^35,36^. The supramarginal gyrus with its strong functional connectivity to OP2 is also crucially involved in self-motion processing^19^. Apart from receiving multisensory inputs, these parieto-insular areas have descending feedback circuits to the vestibular nuclei^35^ and the cerebellum which controls balance, postural control and egomotion perception by its multisensory visual and vestibular inputs to coordinate eye, head and body movements^37^.

Our interaction analysis (GVS x GROUP) revealed stronger activations in insular and cerebellar cortex (Supplementary Table S1) and ROI analysis showed larger activation in the patients’ opercular cortex OP3 and OP4, supramarginal gyrus and inferior parietal lobule when identical GVS stimuli were applied in both groups (Fig. 2). This contradicts previous reports on lower activation to sound-evoked vestibular stimulation in the parieto-insular vestibular cortex in a PPPD precursor condition^17^ which may be related to the specific target of the stimulation technique, i.e., the otoliths and the concomitant stimulation of auditory afferents. By contrasting GVS-evoked brain activation against the sham condition and our recordings in darkness, we diminished visual and somatosensory inputs making it likely that stronger activations in above mentioned brain areas were indeed caused by vestibular stimulation.

We provide some evidence that the stronger activation in multisensory vestibular network is related to the functional impairment of PPPD patients since GVS-evoked neural activity in insular/opercular cortex as well as cerebellar uvula increased with the severity of PPPD (NPQ, ALQ, Fig. 4A + B). This could be linked to (i) altered perceptional thresholds of GVS-induced egomotion, (ii) an exaggerated stimulus-neural amplification in vestibular processing, particularly egomotion encoding brain areas suggesting cortical sensitization or (iii) an exaggerated stimulus-perceptual response, i.e., an abnormal perceptual scaling of identical GVS stimuli.

In line with previous studies^15,30^ patients showed lower GVS perception thresholds. Altered thresholds of GVS-evoked vestibular perception might come from peripheral vestibular hypofunction^32^. However, our PPPD patients had normal quantitative peripheral vestibular function at the time of our brain activation studies. As lower thresholds may impact the magnitude of cortical activation, we introduced threshold dependent GVS intensities (*low* GVS, *high* GVS) to allow comparisons of similar perceptions rather than exclusively identical physical stimulus intensities^32^. Interestingly, the largest group differences were found with *fixed* GVS: GVS with the same physical properties elicited a stronger activation in the multisensory vestibular network that was not regularly obtained by the threshold dependent GVS intensities. Thus, the stronger activation suggests independence from the lower perceptual threshold of egomotion by GVS which is substantiated by two analyses: (i) the missing correlation of the GVS perceptional thresholds with brain activations in the vestibular networks and (ii) the continuing brain activation differences when using the GVS-induced egomotion threshold as a covariate (Supplementary Table S3).

The magnitude of egomotion perception by identical physical energy of vestibular stimulation (*fixed* GVS) differed between groups: patients rated *fixed* GVS higher compared to HC suggesting that the larger activation in insular/opercular cortex and the cerebellum could be the neural substrate for abnormal egomotion, at least for perceived dizziness and abnormal egomotion in PPPD patients during head and body motions (Table 1). The lack of correlations of the GVS-evoked brain activation with the perceived egomotion intensity (Fig. 3A) may be related to the saturation effect both in patients and HC since perceived egomotion intensity did not increase with *high* GVS any longer. As the ratio of perceived egomotion intensity to brain activation by *fixed* GVS did not differ between groups, the larger activation in the patients’ vestibular network areas primarily reflects an abnormal sensory-neural amplification, i.e., neural sensitization, rather than an abnormal sensory-perceptual scaling. Thus, abnormal egomotion perception of PPPD patients seems to result from an abnormal (vs. HC) increase of neural brain response to sensory stimulation (GVS) but not primarily from a perceptual disorder as patients perceive the increased activation of egomotion encoding brain areas proportionally, with no group differences in the ratio of brain activity/rating of perceived egomotion. Abnormal perceptual scaling of sensory stimuli is a mechanism involved in central maladaptive processes in several brain diseases, e.g. central pain syndrome^38^ but little is known about the proportion of individual activation and perception in PPPD. In addition, PPPD patients are prone to perceive egomotion even when non-vestibular stimuli are applied (*sham* GVS); their rating was reasonably low but twice as high compared to HC (14% vs. 7%, Table 1). After adjusting for multiple testing, this distinction was no longer significant (*p* = 0.116). Anticipation may contribute to this amplification of neural responses to GVS but it is important to realize that the larger activation in the patients’ cortical vestibular network was contrasted against the *sham* GVS condition.

There were two regions crucially involved in human egomotion which did not show group differences in our study: the visual area of the cingulate sulcus (CSv) and the cerebellar uvula^19^. This may be related to a threshold dependent activation of these regions. We used a lower GVS intensity (1.3 mA in *fixed* GVS and nearly 2 mA in *high* GVS) compared to a related study applying 3 mA^19^. This needs to be taken into consideration in future egomotion studies by GVS without ignoring the saturation effect with *high* GVS (no further increase in egomotion perception).

Additional data support the notion that the stronger activation in the patients’ multisensory vestibular network is related to their functional impairment: GVS-evoked activation in the insula increased with the patients’ postural instability (postural sway velocity) in the least challenging standing condition (eyes open, firm support) in which patients typically show abnormal postural control (Fig. 4C)^15,39,40^.

Interestingly, in the absence of significant group differences, GVS-evoked activation in the primary visual cortex (V1, V2) and CSv increased with the levels of PPPD-related functional impairment (NPQ and ALQ). This is in line with a related study^10^ with patients suffering from PPPD precursor conditions showing an increase of visual cortex activity (V1 – V3) by visual motion stimuli with the dizziness handicap inventory (DHI), an unspecific dizziness disability questionnaire, not designed for PPPD. In combination with abnormal visual cortex activation in response to visual virtual-reality rollercoaster simulation^23^ and visual motion stimuli^25^ this increased excitability has been suggested as the neural substrate of the visual dependency in PPPD^41,42^. The classification criteria of PPPD include worsening of postural-perceptual dizziness by complex visual motion stimuli. The network engaged in visual motion detection (MT/V5) and the precuneus was reported to be less connected in patients with postural phobic vertigo^25^. Visual cortex activation by our GVS did not correlate with the visual motion coherence thresholds which is in line with studies on HC showing a reduced activation in visual areas during a motion coherence task^43^. Alternatively, aggravation of PPPD symptoms by complex visual stimuli might not necessarily come from increased reliance on visual processing for postural control but from increased coherent motion detection thresholds^14^.

Anxiety, depression and personality traits like neuroticism are thought to be risk factors for the development of PPPD^6,15,24^. Using virtual reality rollercoaster motion stimuli, neural activation in the inferior frontal gyrus and its functional connectivity to the visual cortex of PPPD patients increased with their neuroticism values possibly affecting visual control of postural perception^23^. Vestibular stimulation (GVS) in our PPPD patients evoked activation in the visual cortex (V1, V2, V5) which decreased with the levels of extraversion trait (NEO-FFI, Supplementary Table S4). It remains to be investigated whether these trends are related to the physiological inverse inhibitory visual-vestibular interaction^44^ as a multisensory mechanism for appropriate self-motion perception. This would require combined visual vestibular stimulation in PPPD.

### Limitation of the study

Our data are restricted to PPPD patients with normal vestibular function at the time of recording. Since many patients develop PPPD secondary to a previous, often still persisting vestibular disorder our conclusions are confined. However, this study is obligatory to rule out that abnormal neural brain activation patterns in secondary PPPD are co-determined by the vestibular disorder itself.

## Conclusion

The larger activation by GVS in the patients’ multisensory cortical vestibular network seems to reflect a sensitization to vestibular stimuli eliciting egomotion perception, as the identical GVS stimuli elicit larger ratings responses in PPPD. It increases with levels of PPPD disability but it is not the consequence of the consistently lower egomotion perception thresholds.

## Methods

### Participants

We examined 28 patients (female [f]: 19) and 28 age- and gender-matched (f: 16) HC in this study. Patient recruitment took place in the University Centre for dizziness and vertigo in Lübeck, Schleswig-Holstein. Only patients who met the diagnostic PPPD criteria of the Bárány Society as well as a disease duration for at least three months were included^1^. Exclusion criteria included cerebral lesions (i.e., stroke, hemorrhage or trauma), substance abuse including regular sedating medication, severe psychiatric disorders (i.e., depression, anxiety disorder), visual impairment as well as ongoing comorbidities: migraine, vestibulopathy, benign paroxysmal postural vertigo, tinnitus or acute hearing loss. All participants had at least a school education of nine years and no language barrier. For characterization, all participants filled out seven questionnaires at home prior to their investigation: the EQ-5D-3 L Questionnaire to determine to current quality of life^45^ the NEO-Five-Factor Inventory (NEO-FFI)^46^ the Hospital Anxiety and Depression Scale (HADS)^47,48^ the short form of the Motion Sickness Susceptibility Questionnaire (MSSQ)^49^ the Niigata PPPD Questionnaire (NPQ)^50^ the Athens-Lubeck-Questionnaire (ALQ)^51^ for PPPD-Subtyping and the Edinburgh Handiness Questionnaire^52^. One HC and four patients were ambidextrous, one patient was left-handed.

Apart from a thorough bedside neurologic examination, all participants were investigated using video-based quantitative head impulse test via the EyeSeeCam^®^ HIT System (Autronics, Hamburg, Germany), subjective visual vertical (normal reference: ± 2.5°) as well as ocular vestibular evoked myogenic potentials prior to the study. For further details see^14^. Five patients had a previous vestibular episode of paroxysmal positional vertigo or vestibular neuritis but none of them or the other participants showed abnormal values at the time of MRI recordings.

The study protocol was conducted in accordance with the Declaration of Helsinki and its later amendments and approved by the local Ethics Committee of the University of Lübeck (AZ 17–036, AZ 21–098). Written informed consent was obtained from all participants.

### Galvanic vestibular stimulation (GVS)

We used a DS5 model (Digitimer Ltd., Letchworth Garden City/UK), for bilateral mastoid stimulation with skin contact electrodes by EasyCap GmbH (Herrsching, Germany). The stimulator and electrodes have been approved and used in previous studies^15,53,54^. First, the skin surface over mastoids was cleaned and pre-treated with a local anesthetic (Anesderm^®^ lotion, Pierre Fabre Dermo-Kosmetik GmbH, Freiburg/Germany) for at least 45 min to avoid potential nociceptive stimulation caused by GVS stimulation. After removing the anesthetic, contact electrodes with commercial contact paste were affixed bilaterally. We subsequently determined individual vestibular thresholds of egomotion perception by starting stimulation at 0.5 mA, moving up or down depending on the participant’s sensory threshold. Each stimulation lasted ten seconds with 1 Hz alternating stimulation. The ramp stimulus profile hampered sharp transients at stimulus onset and offset (ramp onset and offset of 100 ms duration) with a stimulation plateau of 300 ms. We used a ramp profile for GVS stimulation, since the main Blood-Oxygenation-Level Dependent (BOLD) effect during fMRI is seen during on- and offset of the stimulation^53,55^. Participants indicated perceived GVS-evoked medio-lateral egomotion by hand movements. Thresholds were secured by varying stimulus intensity until the threshold was proved to be stable. Four stimulus intensities were used: (i) a stimulus intensity (at 1.3 mA) applied independent of the individual threshold (*fixed* GVS*)*, (ii) *a* GVS *of* 0.5 mA *(low* GVS*)* and 1.5 mA *(high* GVS*)* above the individual threshold, (iii) a short stimulus ramp of 100 ms with the *fixed* GVS intensity followed by 400 ms without stimulation that did not elicit egomotion perception (*sham* GVS*)*. Each stimulus lasted 12 s and was applied to each participant before image recording.

### Image acquisition

Structural and functional MR imaging was performed at the Center of Brain, Behaviour and Metabolism (CBBM) Core Facility Magnetic Resonance Imaging using a 3-T Siemens Magnetom Skyra scanner equipped with a 64-channel head-coil. Functional images were acquired applying a single-shot gradient-recalled echo-planar imaging (GRE-EPI) sequence sensitive to BOLD contrast (TR = 1020 ms; TE = 29 ms; flip angle = 70°; in-plane resolution 3 × 3 mm²; 192 × 192 mm² field of view; 60 transversal slices; 3 mm slice thickness; simultaneous multi-slice factor 4); 4 runs of each 516 volumes were recorded. Additionally, structural images of the whole brain using a 3D T1-weighted MP-RAGE sequence were acquired (TR = 1900 ms; TE = 2.44 ms; TI = 900 ms; flip angle 9°; 1 × 1 × 1 mm³ resolution; 192 × 256 × 256 mm³ field of view; acquisition time 4.5 min). T1-weighted images were inspected for pathological findings by a neuroradiologist. Head movements were minimized using ear pads (Multipad Ear, Pearltec Technology AG, Schlieren [CH]). Ear plugs were used for noise cancellation. During recordings, eye movements were recorded via a video based eyetracker (Eyelink 1000 Plus, 1000 Hz sampling rate, SR Research Ltd., Ottawa, ON/Ca) to monitor eye movements.

### Stimuli conditions during functional imaging acquisition

Stimuli conditions consisted of the 4 GVS stimulus intensities with 4 conditions in total. Each condition lasted 12 s and was replicated 3 times in pseudorandomized order with a 10-second break in-between. After each condition, a rating of dizziness and unpleasantness regarding the GVS stimulus was achieved (4 s time for a rating) to accomplish a 9-minute (526 s) run in total. Overall, 4 runs were completed with a 2-minute break between each run. Conditions, breaks and ratings were shown on a monitor (NordicNeuroLab LCD Monitor, 32-inch diameter, screen resolution: 1920 × 1080 pixel, 60 Hz refresh rate) 1.34 m behind the participant. Each condition was presented with a central red dot (diameter 0.5°). Rating was accomplished by using a joystick on the right-hand side (Tethyx, HHSC-JOY-5, Current Designs Inc., Philadelphia (PA)). In-between breaks (no stimulation, no rating) were presented with a central orange dot on the monitor. Stimuli were produced by using Psychophysics Toolbox Version 3 (PTB-3^56^, implemented in Matlab 2022b (The Mathworks, Natick, MA).

### Experimental procedure

The total imaging acquisition time was 65 min. Participants were positioned in standard procedure with a special eyetracking mirror placed above their face to see the monitor behind them. The lights were switched off during examination. The T1-weighted image and the resting state condition was recorded in the first 15 min with a short break afterwards for affixing the electrodes and the assessment of the individual threshold. A joystick was placed on the right-hand side to rate manually egomotion perception intensity following each experimental stimulus condition on a visual-analogue-scale from 0 (no) to 100 (most intensive egomotion) presented on the monitor. Participants were carefully instructed to only rate the effect of the vestibular stimulation without rating other impacts like darkness, sounds or possible somatosensory or nociceptive input. Experimental conditions (e.g., looking on a screen via a mirror, the use of the joystick) were explained and practiced beforehand.

### Behavioral and perceptual variables

Brain activation parameters during GVS in patients and HC were correlated with the following behavioral and perceptual parameters obtained outside the scanner: visual motion perception threshold, passive egomotion threshold and quantitative posturography which have been published elsewhere^14,30^ and therefore only explained in short. Visual motion perception threshold was accomplished via random dot kinematograms with a percentage of dots moving in the same direction. Participants detected this visual motion coherence with increasing difficulty (less coherently moving dots) until they could no longer detect coherent motion. The individual visual motion perception threshold was determined as the percentage of coherently moving dots still correctly identified. Passive egomotion thresholds were obtained by passive horizontal rotation on a turntable. Participants sat on the turntable with a response button in the hand to respond as soon as they recognized egomotion by passive movement of the chair being constrained from visual and auditory cues. Passive egomotion perception was determined in °/s at the time of response. Posturography included a number of conditions which we combined differently: participants stood either on a firm or foam platform with closed or open eyes while they received GVS in some conditions. We obtained the postural sway speed (mm/s) as a parameter for postural control.

### Prepocessing and analysis

MRI-preprocessing and first level analysis were performed using the SPM12 software (Wellcome Trust Centre for Neuroimaging, London/UK) implemented in Matlab 2022b (The Mathworks Inc., Natick/MA). Slice timing correction, motion correction by rigid body spatial transformation to the mean functional image of each dataset (individual head motion was < 3 mm or 3°, minimum to maximum), spatial normalization to a standard template (Montreal Neurological Institute, MNI), resampling to 3 × 3 × 3 mm³ and spatial smoothing (6 mm full width half maximum Gaussian kernel) was completed. We estimated individual head motion by the six realignment parameters, i.e., three rotational and three translational movements with respect to the first image in the EPI series. Functional MRI time series were modeled using a general linear model (GLM). The GLM included regressors for the start of every type of stimulation trial convolved with the canonical hemodynamic response function of SPM12. The six motion parameters were used as covariates in the GLM.

We commenced the following analysis by performing whole brain analysis with a flexible factorial design^57^ for the factors GROUP (patients, HC) and STIMULUS (*low*,* fix*,* high* GVS) against *sham* GVS to rule out brain activity by the stimulation itself as well as the interaction between GROUP and STIMULUS. We applied clusters-wise calculation with limits at a p-value < 0.05 with family wise error (FWE) correction for multiple testing. Activations were anatomically localized with the Automated Anatomical Labeling (AAL^58^), and cytoarchitectonic probability maps^59,60^. Regions of interest (ROI, taken from both brain sides) were defined by using SPM Anatomy Toolbox (Version 3.0,^59^). For ROIs with significant different activations between groups, we calculated the ratio between ROI activation (t-values) and GVS-evoked egomotion perception (median of rating percentage) for each stimulus condition and compared it between groups.

### Statistical analysis

Statistical analyses were performed with SPSS (22.0.0.2; IBM Corp., Somer NY). For comparison of groups and stimulus, we used analysis of variance (ANOVAs) and post hoc t-tests which were Bonferroni corrected for multiple testing. Sphericity requirement was violated in several ANOVAs; therefore p-values are reported with Greenhouse-Geisser correction. Statistical comparisons were performed parametric unless stated otherwise. For correlation analysis with questionnaires and disease parameters, Spearman-Rho nonparametric correlation was used. Statistical differences were regarded as significant for values *p* < 0.05. Data reported indicate mean values (M) and standard deviation (SD) unless otherwise stated.

## Electronic supplementary material

Below is the link to the electronic supplementary material.

Supplementary Material 1

## Data Availability

Raw imgaing, behavioral and questionnaire data are not publicly available to preserve individuals’ privacy. The data are, however, available from the authors upon reasonable request.
