# Supplementary material for: Functional brain activity in persistent postural-perceptual dizziness (PPPD) during galvanic vestibular stimulation reveals sensitization in the multisensory vestibular cortical network
Source: Sci Rep. 2025 Jul 27;15:27355. doi: 10.1038/s41598-025-11529-2 (PMC12301453; doi:10.1038/s41598-025-11529-2)
Supplement: Supplementary file 1 — Supplementary Material 1 [file 41598_2025_11529_MOESM1_ESM.pdf]

Functional brain activity in persistent postural-perceptual dizziness (PPPD) during galvanic vestibular stimulation reveals sensitization in the multisensory vestibular cortical network

Renana Storm[1*], Viktoria Wrobel[1*], Antonia Frings[1],

Andreas Sprenger[1-3], Christoph Helmchen[1,2]

[1] Department of Neurology, University Hospital Schleswig-Holstein, Lübeck, Germany

[2] Center of Brain, Behavior and Metabolism (CBBM), University of Lübeck, Ratzeburger Allee 160, 23562 Lübeck, Germany

[3] Institute of Psychology II, University Lübeck, Germany

* Shared first authorship

Supplementary Data

Results

Imaging results: group differences

Table 1: Group contrasts of brain activation (contrasting GVS vs. sham) of PPPD exceeding HC using different GVS intensities

Cluster	Brain region	Cluster size	x	y	z	t-value
Cluster 1	Cerebellum_4_5_R	491	21	-37	-22	9.92
Cluster 2	Supp_Motor_Area_R	869	3	-7	68	8.81
Cluster 3	Paracentral_Lobule_L	144	-15	-31	74	8.58
Cluster 4	Parietal_Sup_L	188	-24	-61	41	7.79
Cluster 5	Vermis_7	431	-3	-67	-28	7.50

GVS intensities overall (FWE corrected, $p < 0.05$). R=right, L=left.

Cluster	Brain region	Cluster size	x	y	z	t-value
Cluster 1	Lingual_L	787	-18	-76	2	5.49
Cluster 2	Supp_Motor_Area_R	18	3	-7	68	4.96
Cluster 3	Precuneus_R	39	3	-49	53	4.40
Cluster 4	Cerebellum_8_R	39	15	-64	-46	4.80
Cluster 5	Frontal_Inf_Oper_R	15	51	14	38	4.33
Cluster 6	Angular_R	23	39	-55	26	4.46

Low vs. *sham* GVS (small volume correction: $p < 0.001$ uncorrected). R=right, L=left.

Cluster	Brain region	Cluster size	x	y	z	t-value
Cluster 1	Cerebellum_4_5_R	38	27	-43	-28	6.36
Cluster 2	Supp_Motor_Area_R	55	3	-4	68	5.60
Cluster 3	Cerebellum_8_L	33	-3	-67	-31	5.34

Fix vs. *sham* GVS (FWE corrected, $p < 0.05$). R=right, L=left.

Cluster	Brain region	Cluster size	x	y	z	t-value
Cluster 1	Postcentral_R	29	27	-34	68	7.49
Cluster 2	Paracentral_Lobule_L	12	-15	-28	74	6.76
Cluster 3	Cerebellum_4_5_R	32	21	-37	-22	6.59
Cluster 4	Temporal_Mid_R	10	48	-55	8	5.48

High vs. *sham* GVS (FWE corrected, $p < 0.05$). R=right, L=left.

Region of interest (ROI) analyses

Table 2: ROI labels and their corresponding name

ROI label	corresponding name
Supramarginal gyrus	Supramarginal gyrus
IPL PF	Inferior parietal lobe
OP 1−4	Parietal operculum, areas 1−4
Insula Ig1+2	Part 1 and 2 of the granular insula
CSv	Visual area of the cingulate sulcus
V1−V5	Visual cortex, areas 1−5
Uvula	Uvula cerebelli
Vermis VII	Lobule VII of the vermis cerebelli

Abbreviation: ROI = region of interest.

Table 3: Brain activation (ROI) in patients and HC independent of the individual GVS threshold

ROI label	group comparison	Covariate GVS threshold
Left supramarginal gyrus	t(54) = 2.41, p = 0.019	t(54) = -0.88 p = 0.385
Left OP4	t(54) = 2.08, p = 0.042	t(54) = 0.06, p = 0.955
Right insula OP 3	t(54) = 2.09, p = 0.042	t(54) = 0.13, p = 0.897
Right IPL PF	t(54) = 4.19, p < 0.001	t(54) = -1.78, p = 0.081
Vermis VII	t(54) = 2.39, p = 0.021	t(54) = -0.65, p = 0.518

Abbreviations: GVS = galvanic vestibular stimulation, HC = healthy control subjects, ROI = region of interest.

Table 4: Brain activation (ROI) in the visual cortex by different GVS intensities decreases with the extraversion trait of the NEO-FFI

ROI	fix GVS	low GVS	high GVS
left V1	**p = 0.007**, r = -0.50	p = 0.276, r = -0,21	**p = 0.017**, r = -0.45
right V1	**p = 0.009**, r = -0.48	p = 0.255, r = -0.22	**p = 0.017**, r = -0.45

left V2	**p = 0.013**, r = -0.46	p = 0.355, r = -0.18	**p = 0.017**, r = -0.45
right V2	**p = 0.017**, r = -0.45	p = 0.243, r = -0.23	**p = 0.016**, r = -0.45
left V5	**p = 0.007**, r = -0.50	p = 0.133, r = -0.29	p = 0.114, r = -0.31
right V5	p = 0.063, r = -0.36	p = 0.252, r = -0.22	p = 0.557, r = -0.12

Brain activity in visual cortex areas during *fix* and *high* GVS in PPPD patients correlates with the extraversion trait of the NEO-FFI. P-value and correlation coefficient for ROI activation are listed for the different GVS intensities (*fix*, *low*, *high*). Significant p-values are marked bold. Abbreviations: GVS = galvanic vestibular stimulation, NEO-FFI = NEO-Five-Factory Inventory, ROI = region of interest.
